# Optogenetic tools for inducing organelle membrane rupture

**DOI:** 10.1016/j.jbc.2025.108421

**Published:** 2025-03-18

**Authors:** Yuto Nagashima, Tomoya Eguchi, Ikuko Koyama-Honda, Noboru Mizushima

**Affiliations:** Department of Biochemistry and Molecular Biology, Graduate School and Faculty of Medicine, The University of Tokyo, Tokyo, Japan

**Keywords:** membrane rupture, Bcl-2-associated X protein (BAX), light-oxygen-voltage-sensing 2 (LOV2) domain, optogenetics, lysosomal membrane permeabilization (LMP), mitochondrial outer membrane permeabilization (MOMP)

## Abstract

Disintegration of organelle membranes induces various cellular responses and has pathological consequences, including autoinflammatory diseases and neurodegeneration. Establishing methods to induce membrane rupture of specific organelles is essential to analyze the downstream effects of membrane rupture; however, the spatiotemporal induction of organelle membrane rupture remains challenging. Here, we develop a series of optogenetic tools to induce organelle membrane rupture by using engineered Bcl-2-associated X protein (BAX), which primarily functions to form membrane pores in the outer mitochondrial membrane (OMM) during apoptosis. When BAX is forced to target mitochondria, lysosomes, or the endoplasmic reticulum (ER) by replacing its C-terminal transmembrane domain (TMD) with organelle-targeting sequences, the BAX mutants rupture their targeted membranes. To regulate the activity of organelle-targeted BAX, the photosensitive light-oxygen-voltage-sensing 2 (LOV2) domain is fused to the N-terminus of BAX. The resulting LOV2-BAX fusion protein exhibits blue light-dependent membrane-rupture activity on various organelles, including mitochondria, the ER, and lysosomes. Thus, LOV2-BAX enables spatiotemporal induction of membrane rupture across a broad range of organelles, expanding research opportunities on the consequences of organelle membrane disruption.

Eukaryotic cells are compartmentalized by organelle membranes, and the disintegration of these membranes triggers numerous biological responses and is associated with various diseases. Excessive release of mitochondrial intermembrane proteins, such as cytochrome *c*, from ruptured mitochondria activates apoptosis signaling through the activation of caspases (1, 2). In cases of minor membrane permeabilization that are insufficient to cause cell death, activated caspases may induce DNA damage and promote oncogenesis (3). Mitochondrial membrane rupture also causes leakage of mitochondrial DNA (mtDNA) (4). Cytoplasmic mtDNA is sensed by cyclic GMP-AMP synthase, activates stimulator of interferon genes (STING), and subsequently initiates inflammatory signaling. These downstream processes are involved in cancers, autoimmune diseases, and neurodegenerative disorders (5, 6). Lysosomal membrane rupture also leads to cytotoxicity *via* leakage of hydrolysis enzymes into the cytosol (7). Because of the undesirable consequences of membrane disintegration, cells have protective mechanisms, including autophagy-mediated clearance (8, 9, 10), and the endosomal sorting complex required for transport (ESCRT) or annexin-dependent repair of ruptured organelles (11, 12). Organelle membrane rupture can also occur under normal physiological conditions. For instance, in lens fiber cells, organelle membranes are first slightly damaged by unknown mechanisms during development and can then be fully degraded by phospholipase A/acyltransferase (PLAAT) family phospholipases, leading to lens transparency (13).

To analyze the molecular responses to organelle membrane rupture and their eventual pathophysiological consequences, methods to induce the rupture of specific organelle membranes are essential. However, at present, spatiotemporal induction of membrane rupture remains a challenge. Although chemical compounds such as B cell lymphoma-2 (Bcl-2) inhibitors and l-leucyl-l-leucine methyl ester (LLOMe) are widely used to rupture the mitochondrial and lysosomal membranes, respectively (14, 15), they cannot induce membrane rupture in specific cells within a tissue or in specific organelles of a single cell. In addition, chemicals that rupture membranes of the endoplasmic reticulum (ER), peroxisomes, or the Golgi apparatus are not yet available. Several studies have reported optogenetic tools to damage organelles (16, 17, 18). Although these tools can spatiotemporally induce membrane rupture, their targets are limited to mitochondria alone (16, 17) or both mitochondria and peroxisomes (18).

Bcl-2-associated X protein (BAX) is a Bcl-2 family protein that mediates permeabilization of the outer mitochondrial membrane (OMM) during apoptosis (19). BAX is classified as a tail-anchored protein that possesses a single transmembrane domain (TMD) in its C-terminal region, followed by a short C-terminal element. In its inactive state, the C-terminal region of BAX is concealed within the N-terminal soluble domain, resulting in BAX being retained in the cytosol (20). Upon receiving apoptotic stimuli, the C-terminal region of BAX becomes exposed, and BAX is then inserted into the OMM, where BAX, along with another proapoptotic protein, Bcl-2 homologous antagonist/killer (BAK), undergoes oligomerization to form a membrane pore (21). It is reported that artificial targeting of BAX or BAK to mitochondria or the ER results in leakage of their luminal contents (22).

In this study, by engineering BAX, we developed a series of optogenetic tools wherein photoactivatable BAX is specifically targeted to the membranes of distinct organelles. Using these tools, we were able to rupture the mitochondrial, lysosomal, and ER membranes in a light-dependent manner.

## Results

### Organelle-targeted BAX ruptures the mitochondrial, lysosomal, and ER membranes

**Figure 1:**
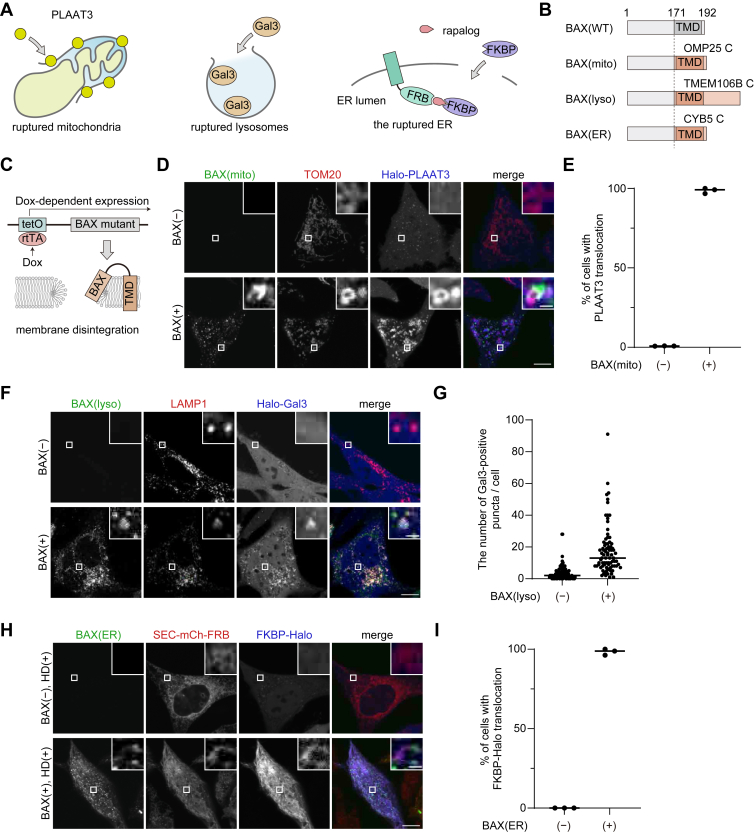
**Forced targeting of BAX induces organelle membrane rupture**. *A*, reporters used to detect organelle membrane rupture. Each reporter translocates from the cytosol to its target organelles upon membrane rupture. *B*, construction of BAX mutants. The C-terminal region of BAX was replaced with that of OMP25, TMEM106B, or CYB5. *C*, regulation of BAX expression using a Tet-On system. A reversed tetracycline transactivator (rtTA) binds to tetracycline operator elements (tetO) and induces expression of the BAX mutants in a doxycycline-dependent manner. The BAX mutants localize to target organelles and rupture the membranes. *D*, HeLa cells expressing TOM20-mRFP and Halo-PLAAT3(C113S) without (*upper panels*) or with (*lower panels*) Tet-ON GFP-BAX(mito) were treated with doxycycline for 1 day in the presence of Q-VD-Oph. The HaloTag ligand conjugated with SaraFluor 650T was added 10 min before observation. Scale bars, 10 μm (*main panels*), 1 μm (*inset panels*). *E*, the percentage of cells showing accumulation of Halo-PLAAT3(C113S)-positive mitochondria among total cells (BAX (−)) or EGFP-positive cells (BAX (+))in (*D*). Horizontal lines indicate the group means, and each dot indicates the data from one of the three independent experiments. At least 100 cells were analyzed in each experiment. *F*, HeLa cells expressing LAMP1-mRFP and Halo-Gal3 without (*upper panels*) or with (*lower panels*) Tet-ON GFP-BAX(lyso) were treated with doxycycline for 2 days in the presence of Q-VD-Oph. HaloTag SaraFluor 650T ligand was added 10 min before observation. Scale bars, 10 μm (*main panels*), 1 μm (*inset panels*). G, quantification of the number of Halo-Gal3 puncta per cell in (*F*). *Horizontal lines* indicate the group means, and each dot indicates the data from each cell. N = 80, 80 cells, respectively. *H*, HeLa cells expressing SEC61B-mCherry-FRB (SEC-mCh-FRB) and FKBP-Halo without (*upper panels*) or with (*lower panels*) Tet-ON GFP-BAX(ER) were treated with doxycycline for 1 day in the presence of Q-VD-Oph. The cells were treated with the heterodimerizer (HD) and HaloTag SaraFluor 650T ligand for 10 min. Scale bars, 10 μm (*main panels*), 1 μm (*inset panels*). *I*, the percentage of cells showing accumulation of FKBP-Halo on the ER membrane in (*H*). Horizontal lines indicate the means, and each dot indicates the data point from one of the three independent experiments. At least 80 cells were analyzed in each experiment.

To establish inducible organelle rupture systems, we first prepared reporters to detect membrane rupture of various organelles. As a marker for mitochondrial membrane rupture, HaloTag (hereafter referred to as Halo)-PLAAT3(C113S), a catalytically inactive mouse PLAAT3 mutant, was used (Fig. 1*A*). PLAAT3 is a lipase that translocate from the cytosol to damaged organelles such as mitochondria (though its mechanism remains unclear) (13). As a marker for lysosomal membrane rupture, we used galectin-3 (Gal3) (Fig. 1*A*), a cytosolic protein that accumulates in ruptured lysosomes by binding to glycosylated proteins along the inner surface of the lysosomal membrane (23). To visualize the ruptured ER, we utilized the FK506-binding protein (FKBP) and FKBP-rapamycin binding protein (FRB) tags, which form a heterodimer in the presence of the rapamycin derivative A/C heterodimerizer (hereafter simply referred to as the heterodimerizer) (24). FRB was expressed in the ER lumen by fusing it to the C-terminus of SEC61B, while FKBP-Halo was expressed in the cytosol. Cytosolic FKBP bound to FRB and accumulated in the ER lumen, depending on both of the heterodimerizer and the rupture of ER membranes (Fig. 1*A*). To verify the proper orientation of SEC61B-mCherry-FRB, mCherry was immunostained following differential permeabilization. Anti-mCherry antibody signals were detected only in Triton X-100-permeabilized cells, where both plasma and organelle membranes were permeabilized, but not in digitonin-permeabilized cells, where only the plasma membrane was permeabilized (Fig. S1). This result confirmed that the C-terminus of SEC61B-mCherry-FRB is in the ER lumen, although its insertion pathway might differ from that of native SEC61B.

Then, we tested whether BAX induced rupture of organelle membranes when it was forced to target mitochondria, lysosomes, and the ER by replacing its C-terminal region with that of respective organelle-specific proteins (Fig. 1*B*). Because organelle membrane rupture may cause cell death, these BAX mutants were inducibly expressed in HeLa cells in a doxycycline-dependent manner (Fig. 1*C*). Cells were cultured in the presence of quinoline-Val-Asp-difluorophenoxymethylketone (Q-VD-Oph), a caspase inhibitor, to block the cell death signaling pathway.

To localize BAX to mitochondria, we replaced the C-terminal region of BAX(171–192) with that of the mitochondrial tail-anchored protein OMP25(109–145), referred to as BAX(mito). When EGFP-BAX(mito) was not expressed, Halo-PLAAT3(C113S) was mostly diffused throughout the cytoplasm with some small punctate structures likely representing peroxisomes, as previously reported (25) (Fig. 1*D*). After induction of EGFP-BAX(mito) expression by doxycycline, EGFP-BAX(mito) was successfully recruited to TOM20-positive OMM. Subsequently, Halo-PLAAT3(C113S) accumulated on these mitochondria in almost all EGFP-BAX(mito)-expressing cells, suggesting that the mitochondrial membranes were ruptured (Fig. 1, *D* and *E*).

To localize BAX to lysosomes, we initially utilized the C-terminal regions of VAMP7 and VAMP8, lysosomal tail-anchored SNARE proteins; however, these constructs failed to localize to lysosomes. We then constructed BAX(lyso), in which the C-terminus of BAX was replaced with that (amino acids 90–274) of TMEM106B, a single-pass lysosomal membrane protein whose N-terminus faces into the cytosol (26). Following doxycycline treatment, EGFP-BAX(lyso) was recruited to the LAMP1-positive lysosomes, and Halo-Gal3 was translocated from the cytosol to the lysosomes (Fig. 1, *F* and *G*). These data suggest that lysosomal membranes were ruptured by BAX(lyso).

We also generated BAX(ER) by replacing the C-terminus of BAX with the C-terminus (amino acids 95–134) of CYB5, an ER transmembrane protein, to localize BAX to the ER. Upon doxycycline treatment, EGFP-BAX(ER) localized to the ER (Fig. 1*H*). When EGFP-BAX(ER) was not expressed, FKBP-Halo was diffusely distributed throughout the cytosol, even in the presence of the heterodimerizer. After EGFP-BAX(ER) expression and heterodimerizer treatment, FKBP-Halo efficiently accumulated on the ER membranes (Fig. 1, *H* and *I*), indicating that EGFP-BAX(ER) induced ER membrane rupture.

These data indicate that forced targeting of BAX to organelle membranes induces membrane rupture regardless of organelle type.

### Tethering BAX to organelle membranes induces mitochondria-specific membrane rupture

**Figure 2:**
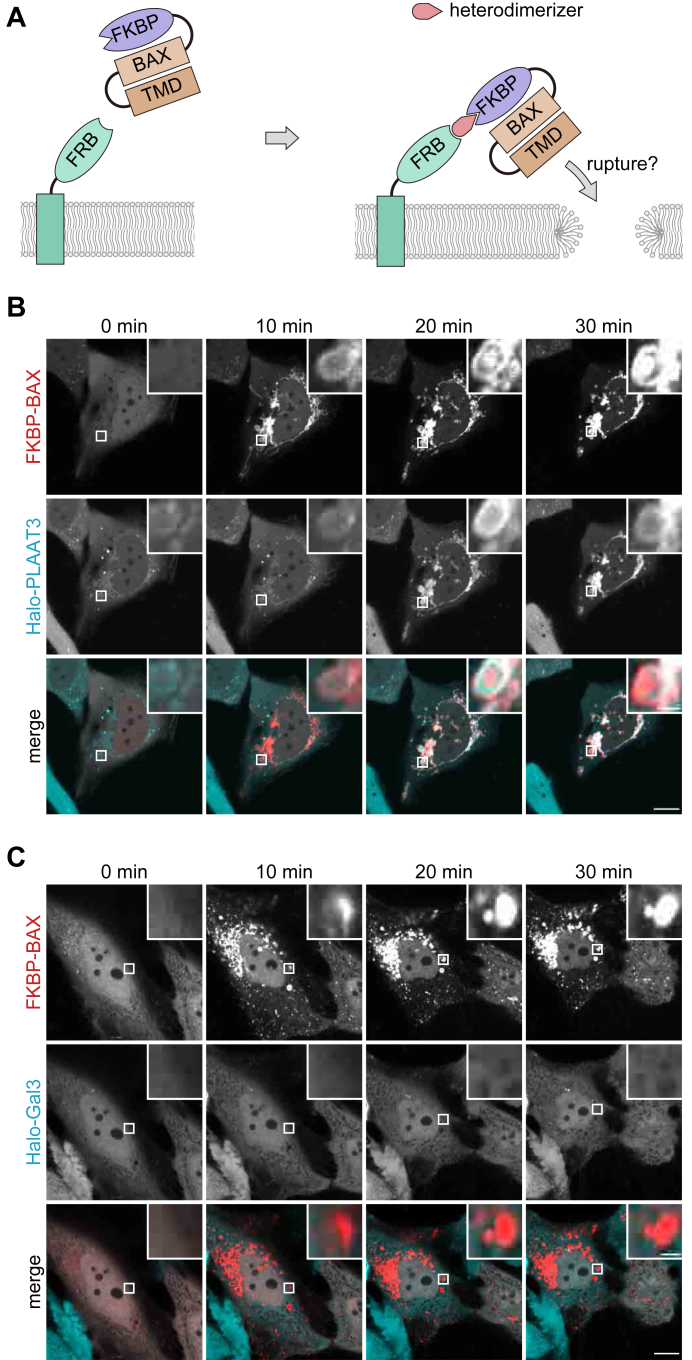
**Membrane tethering of BAX causes rupture of the outer mitochondrial membrane but not the lysosomal membrane**. *A*, a schematic representation of the ectopic tethering of BAX mutants to organelle membranes *via* the FKBP and FRB tags. FKBP-mCherry-BAX(S184E) in the cytosol and FRB on organelle membranes form a heterodimer in the presence of heterodimerizer, bringing BAX into close proximity to the organelle membranes. *B*, HeLa cells expressing FKBP-mCherry-BAX(S184E), TOM20(1-69)-FRB, and Halo-PLAAT3(C113S) were treated with heterodimerizer and Q-VD-Oph, which were added at 0 min. HaloTag SaraFluor 650T ligand was added 10 min before observation. Scale bars, 10 μm (*main panels*), 1 μm (*inset panels*). *C*, HeLa cells expressing FKBP-mCherry-BAX(S184E), LAMP1-FRB, and Halo-Gal3 were treated with heterodimerizer and Q-VD-Oph, which were added at 0 min. HaloTag SaraFluor 650T ligand was added 10 min before observation. Scale bars, 10 μm (*main panels*), 1 μm (*inset panels*).

Although the doxycycline-based system successfully induced organelle membrane rupture, its transcription-dependent nature was relatively slow. To overcome this limitation, we sought to introduce an optogenetic system. Previous studies have demonstrated that tethering of cytoplasmic BAX to the mitochondrial membranes using optogenetic tags can be used to induce mitochondrial membrane rupture (16, 17). In this system, photoactivable dimerization modules from *Arabidopsis* cryptochrome-interacting basic-helix-loop-helix 1 (CIB) and cryptochrome 2 (CRY2) are fused to TOM20 and BAX (with mutation S184E to prevent background mitochondrial targeting), respectively. Photo-induced dimerization of CRY2 and CIB tags tethers BAX to the OMM, inducing its rupture. To test whether a similar method can be applied to the inducible rupture of other organelles, we modified the BAX translocation system using the FKBP and FRB tags (Fig. 2*A*).

As expected, FKBP-mCherry-BAX(S184E) was diffusely distributed throughout the cytoplasm in the absence of the heterodimerizer. Upon heterodimerizer treatment, FKBP-mCherry-BAX(S184E) immediately accumulated on mitochondria in HeLa cells expressing TOM20-FRB (Fig. 2*B*). Halo-PLAAT3(C113S) also accumulated on mitochondria, indicating that anchoring of BAX induced mitochondrial rupture (Fig. 2*B*). However, when FKBP-mCherry-BAX(S184E) was recruited to lysosomes *via* LAMP1-FRB, no Halo-Gal3 accumulation was observed in lysosomes (Fig. 2*C*). These data suggest that tethering of BAX is sufficient to induce membrane rupture only in mitochondria and not in lysosomes, prompting us to explore alternative methods to induce membrane rupture.

### Mitochondria-targeted LOV2-BAX induces blue light-dependent OMM rupture

**Figure 3:**
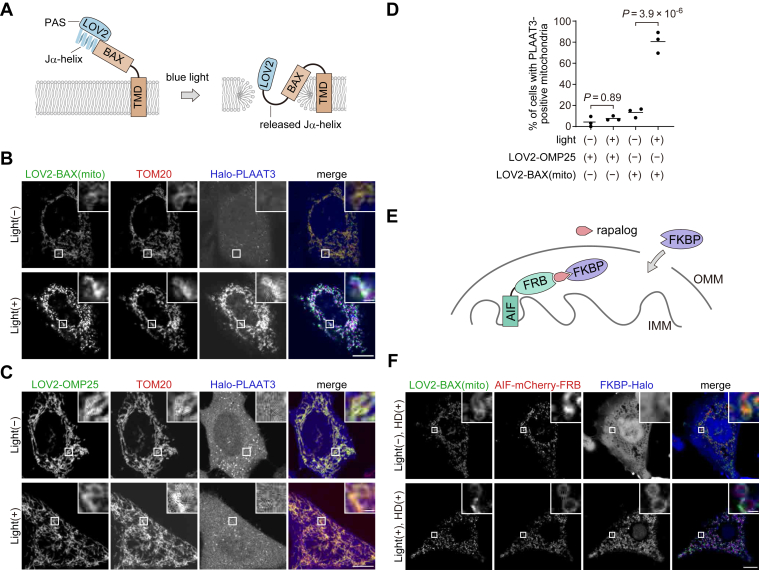
**The LOV2 domain enables photo-dependent regulation of the pore-forming activity of BAX on mitochondria.***A*, the strategy used to regulate the pore-forming activity of BAX in a photostimulation-dependent manner. LOV2 attachment inhibits the pore-forming activity of BAX. The Jα-helix of LOV2, connected to the N-terminus of BAX, is released from the PAS domain and unfolded upon blue-light stimulation. This conformational change activates the pore-forming activity of BAX. *B*, GFP-LOV2-BAX(mito), TOM20-mRFP, and Halo-PLAAT3(C113S) were expressed in HeLa cells. Cells were treated with Q-VD-Oph and HaloTag SaraFluor 650T ligand, stimulated with *blue light* for 60 min, and subsequently incubated for 30 min before fixation. Scale bars, 10 μm (*main panels*), 1 μm (*inset panels*). *C*, GFP-LOV2-OMP25, TOM20-mRFP, and Halo-PLAAT3(C113S) were expressed in HeLa cells. Cells were treated as in (*B*). Scale bars, 10 μm (*main panels*), 1 μm (*inset panels*). *D*, the percentage of cells showing accumulation of Halo-PLAAT3(C113S) on the outer mitochondrial membrane. *Horizontal lines* indicate the group means, and each dot indicates the data point from one of three independent experiments. Differences were statistically analyzed using one-way ANOVA with Tukey's test. At least 70 cells were observed in each experiment. *E*, a schematic illustration of the detection of mitochondrial rupture using AIF(1–90)-mCherry-FRB and FKBP-Halo reporters. Dependent on mitochondrial rupture and the presence of the heterodimerizer, cytosolic FKBP-Halo binds to the FRB tag located in the intermembrane space of mitochondria, resulting in the accumulation of FKBP-Halo in mitochondria. *F*, GFP-LOV2-BAX(mito), AIF(1–90)-mCherry-FRB, and FKBP-Halo were expressed in HeLa cells. Cells were treated with the heterodimerizer (HD), stimulated with *blue light* for 60 min, and subsequently incubated for 30 min before fixation. Scale bars, 10 μm (*main panels*), 1 μm (*inset panels*).

**Figure 4:**
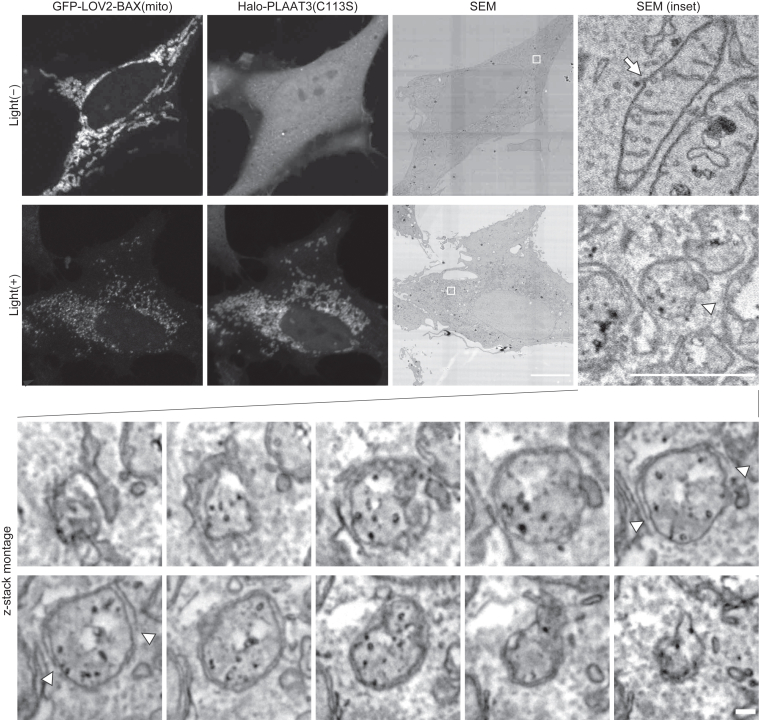
**Electron micrographs of mitochondria ruptured by GFP-LOV2-BAX(mito).** GFP-LOV2-BAX(mito) and Halo-PLAAT3(C113S) were expressed in HeLa cells. Cells were treated with Q-VD-Oph and HaloTag SaraFluor 650T ligand, stimulated with *blue* light for 60 min, subsequently incubated for 30 min before fixation, and analyzed by correlative light and electron microscopy. An arrow indicates a normal mitochondrion and an *arrowhead* indicates a membrane-ruptured mitochondrion (u*pper panels*). Serial sections (25-nm thickness) of a membrane-ruptured mitochondrion are shown (*bottom panels*). Arrowheads in the serial sections indicate the edge of the outer mitochondrial membrane (OMM) of the ruptured mitochondrion. SEM, scanning electron micrograph. Scale bars, 10 μm (main panels), 1 μm (*enlarged panels*), 100 nm (serial sections).

BAX insertion into membranes *via* its modified C-terminus induced membrane rupture of various organelles (Fig. 1). Therefore, we attempted to control the activity of the “membrane-inserted” BAX mutants rather than its translocation. To this end, we used the light-oxygen-voltage-sensing domain 2 (LOV2), a photosensor domain from *Avena sativa* phototropin1 (Fig. 3*A*). The LOV2 domain is composed of the N-terminal Period-ARNT-Singleminded (PAS) domain and the C-terminal Jα-helix, which tightly bind to each other and can inhibit the function of downstream fused proteins through steric hindrance (27). Upon blue light stimulation, the Jα-helix is released from the PAS domain and then unfolded (28). Because of this photo-dependent dynamic conformational change, the LOV2 domain is widely used as a photoswitch to regulate the function of proteins by fusing it to their N-termini (29). By fusing LOV2(N538E), a mutant with reduced background activation (30), to the N-terminus of BAX(mito), we constructed LOV2(N538E)-BAX(3–171)-OMP25, referred to as LOV2-BAX(mito). EGFP-LOV2-BAX(mito) localized onto mitochondria irrespective of blue light stimulation (Fig. 3*B*). In contrast to EGFP-BAX(mito), EGFP-LOV2-BAX(mito) caused only minor accumulation of Halo-PLAAT3(C113S) on mitochondria without blue light stimulation (Fig. 3, *B* and *D*), indicating that LOV2 domain suppresses membrane-damaging activity of BAX. After blue light stimulation for 60 min followed by 30-min incubation, Halo-PLAAT3(C113S) accumulated on the mitochondria (Fig. 3, *B* and *D*). In these cells, EGFP-LOV2-BAX(mito) formed bright puncta on the OMM, probably because EGFP-LOV2-BAX(mito) accumulated at membrane pores (see Discussion). Blue light did not induce translocation of Halo-PLAAT3(C113S) in cells expressing EGFP-LOV2-OMP25 without the BAX cytoplasmic domain, indicating that mitochondrial rupture is not caused by phototoxicity (Fig. 3, *C* and *D*). To confirm mitochondrial rupture, we used previously reported probes based on chemically dimerizable FKBP/FRB domains with slight modifications (3). A Halo-fused FKBP domain and a mitochondria-targeted FRB domain (fused with the mitochondrial anchoring sequence of apoptosis-inducing factor [AIF]) were introduced into HeLa cells expressing EGFP-LOV2-BAX(mito) (Fig. 3*E*). As expected, FKBP-Halo translocated from the cytosol to mitochondria in a manner dependent on blue light stimulation and the heterodimerizer (Fig. 3*F*), supporting the conclusion that EGFP-LOV2-BAX(mito) induces photo-dependent OMM rupture. The OMM rupture by EGFP-LOV2-BAX(mito) was further confirmed by correlative light and electron microscopy. Without blue light, mitochondria showed oval shapes with clear cristae structures (Fig. 4). After blue light stimulation, Halo-PLAAT3(C113S) accumulated on mitochondria, which were swollen with OMM rupture and cristae structure loss (Fig. 4). These data indicate that LOV2-BAX enables blue light-dependent induction of mitochondrial membrane rupture.

### LOV2-BAX induces rupture of lysosomal and ER membranes

**Figure 5:**
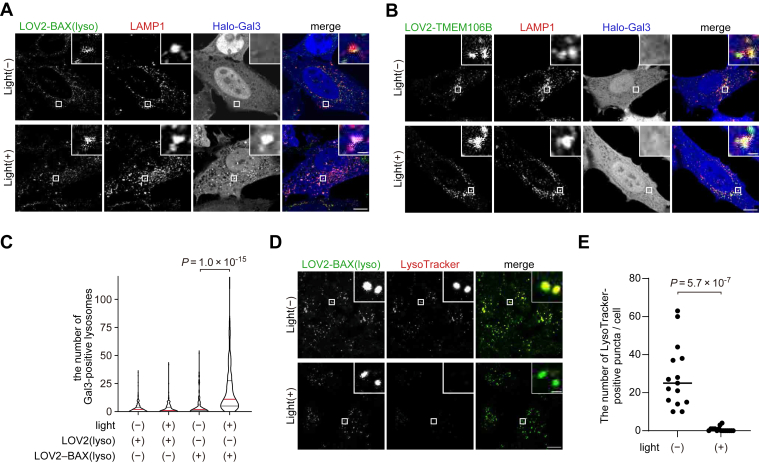
**Photo-regulated lysosome rupture by LOV2-BAX.***A* and *B*, HeLa cells expressing LAMP1-mRFP, Halo-Gal3, and GFP-LOV2-BAX(lyso) (*A*) or GFP-LOV2-TMEM106B (*B*) were treated with Q-VD-Oph and HaloTag SaraFluor 650T ligand, stimulated with *blue* light for 60 min, and subsequently incubated for 30 min before fixation. Scale bars, 10 μm (*main panels*), 1 μm (*inset panels*). *C*, quantification of the number of Halo-Gal3 puncta per cell in (*A* and *B*). Differences were statistically analyzed by one-way ANOVA with Tukey's test. The red lines represent the median, and the black lines represent the quartiles. At least 150 cells were analyzed in each experiment. *D*, HeLa cells expressing GFP-LOV2-BAX(lyso) were stimulated with *blue* light for 60 min, incubated for 30 min in the presence of LysoTracker Red, and then fixed. Scale bars, 10 μm (*main panels*), 1 μm (*inset panels*). *E*, quantification of the number of LysoTracker-positive structures per cell in (*D*). Differences were statistically analyzed by unpaired *t* test. *Horizontal lines* indicate the group means, and each dot indicates the data from each cell.

We tested whether LOV2-BAX induced membrane rupture in organelles other than mitochondria. To induce lysosomal rupture, we first tested EGFP-LOV2(N538E)-BAX(3-171)-TMEM106B(90-274). However, this construct caused high background lysosomal damage independent of blue light. To reduce background activation, we introduced an additional mutation, G528A, which enhances the structural stability of the LOV2 domain under dark conditions (30), and generated EGFP-LOV2(G528A, N538E)-BAX(3–171)-TMEM106B(90–274), referred to as EGFP-LOV2-BAX(lyso). Before blue light stimulation, cells expressing EGFP-LOV2-BAX(lyso) showed only a few Gal3-positive lysosomes (Fig. 5*A*). The number of Gal3-positive puncta increased after photostimulation (Fig. 5, *A* and *C*), indicating that EGFP-LOV2-BAX(lyso) triggers blue light-dependent lysosomal rupture. Photo-dependent lysosomal rupture was not observed in cells expressing LOV2-TMEM106B, which lacks the BAX cytoplasmic domain (Fig. 5, *B* and *C*). To further confirm lysosomal rupture after LOV2-BAX photostimulation, we analyzed signals of LysoTracker, a fluorescent dye that accumulates in lysosomes depending on the proton gradient. In the absence of photostimulation, LysoTracker signals were observed in LOV2-BAX(lyso)-positive structures (Fig. 5*D*), indicating that the lysosomal lumen remained acidic. In contrast, photostimulated cells showed decreased LysoTracker signals (Fig. 5, *D* and *E*), indicating that activation of LOV2-BAX(lyso) induces proton leakage from lysosomes.

**Figure 6:**
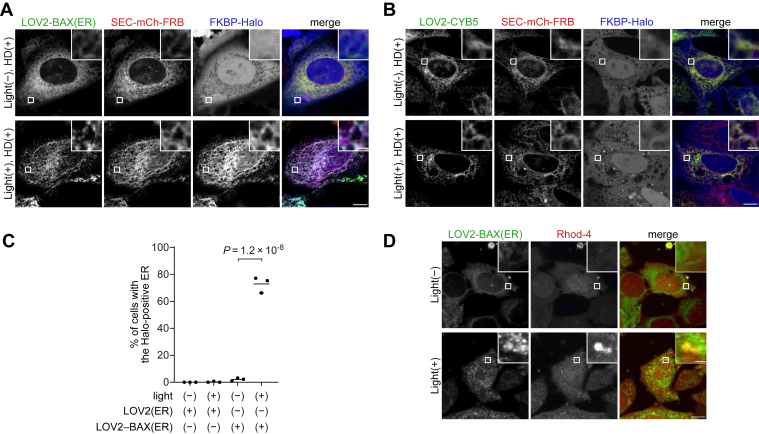
**Photo-regulated ER rupture by LOV2-BAX**. *A* and *B*, HeLa cells expressing SEC61B-mCherry-FRB (SEC-mCh-FRB), FKBP-Halo, and either GFP-LOV2-BAX(ER) (*A*) or GFP-LOV2-CYB5 (*B*) were treated with the heterodimerizer (HD), Q-VD-Oph, and HaloTag SaraFluor 650T ligand, stimulated with blue light for 60 min, and subsequently incubated for 30 min before fixation. Scale bars, 10 μm (*main panels*), 1 μm (*inset panels*). *C*, the percentage of cells showing accumulation of FKBP-Halo on the ER membrane in (*A* and *B*). *Horizontal lines* indicate the means, and each dot indicates the data point from one of three independent experiments. Differences were statistically analyzed using one-way ANOVA with Tukey's test. At least 100 cells were analyzed in each experiment. *D*, HeLa cells expressing LOV2-BAX(ER) were stimulated with *blue* light for 60 min, and subsequently incubated for 30 min in the presence of Rhod-4 before live imaging. Scale bars, 10 μm (*main panels*), 1 μm (*inset panels*).

To induce photo-dependent ER rupture, we constructed EGFP-LOV2(N538E)-BAX(3–171)-CYB5(95–134), referred to as EGFP-LOV2-BAX(ER). EGFP-LOV2-BAX(ER) was expressed in HeLa cells, and ER rupture was then assessed by using the SEC61B-mCherry-FRB and FKBP-Halo reporters. EGFP-LOV2-BAX(ER) colocalized with SEC61B-mCherry-FRB, confirming the ER localization of LOV2-BAX(ER) (Fig. 6*A*). In the presence of the heterodimerizer, FKBP-Halo accumulated in the ER in a manner dependent on both LOV2-BAX(ER) and blue light stimulation (Fig. 6, *A*–*C*). In addition to the ER rupture reporter based on FKBP-FRB, we assessed calcium leakage from the ER. Cells expressing LOV2-BAX(ER) were cultured with or without photostimulation for 1 h, followed by treatment with Rhod-4 AM, a dye that accumulates in the cytosol and increases fluorescence intensity in response to calcium ions. Cells without photostimulation showed diffuse signals of Rhod-4 AM (Fig. 6*D*). In contrast, photostimulated cells showed strong Rhod-4 AM signals around LOV2-BAX(ER)-positive structures (Fig. 6*D*). These data suggest that the activation of LOV2-BAX(ER) causes calcium leakage from the ER.

### LOV2-BAX specifically ruptures their targeted organelles depending on its pore-forming activity

We assessed the involvement of endogenous BAX and BAK in the activity of LOV2-BAX. LOV2-BAX(mito), LOV2-BAX(lyso), or LOV2-BAX(ER) were expressed in cells depleted of endogenous BAX and BAK. Successful knockdown was confirmed by immunoblotting (Fig. S2*A*). Even in cells depleted of endogenous BAX and BAK, LOV2-BAX efficiently induced membrane rupture in the targeted organelles upon photostimulation (Fig. S2, *B*–*D*), suggesting that endogenous BAX and BAK are not required for LOV2-BAX activity.

To determine whether the activity of LOV2-BAX is indeed mediated by its oligomerization and pore-forming capabilities, a G108V mutation was introduced into BAX. This mutation disrupts BAX oligomerization and apoptosis induction (31). The mutated constructs did not rupture their targeted organelle membranes: LOV2-BAX(mito, G108V) did not induce PLAAT3 translocation to mitochondria (Fig. S3*A*), LOV2-BAX(lyso, G108V) did not induce Gal3 translocation to lysosomes (Fig. S3*B*), and LOV2-BAX(ER) did not induce FKBP-Halo translocation to the ER (Fig. S3*C*). These results confirm the importance of the pore-forming activity of BAX.

**Figure 7:**
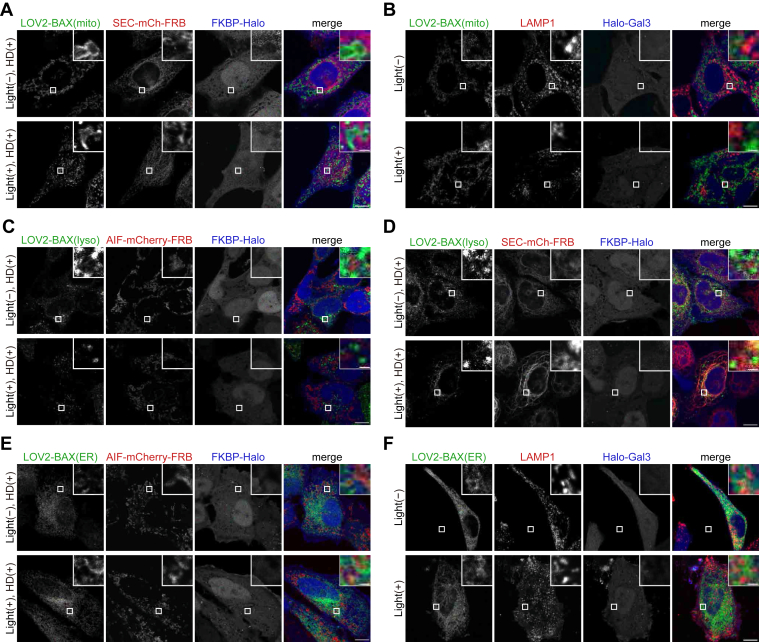
**LOV2-BAX constructs specifically rupture their targeted organelles**. *A* and *B*, HeLa cells expressing LOV2-BAX(mito) and the organelle damage reporters for the ER (SEC61B-mCherry-FRB and FKBP-Halo) (*A*) or for lysosomes (LAMP1-RFP and Halo-Galectin 3) (*B*). Cells were treated with Q-VD-Oph, HaloTag SaraFluor 650T ligand, and the heterodimerizer (only for *A*), stimulated with *blue* light for 60 min, and subsequently incubated for 30 min before fixation. Scale bars, 10 μm (main panels), 1 μm (inset panels). *C* and *D*, HeLa cells expressing LOV2-BAX(lyso) and the organelle damage reporters for mitochondria (AIF-mCherry-FRB and FKBP-Halo) (*C*) or for the ER (SEC61B-mCherry-FRB and FKBP-Halo) (*D*). Cells were treated with Q-VD-Oph, HaloTag SaraFluor 650T ligand, and the heterodimerizer, stimulated with *blue* light for 60 min, and subsequently incubated for 30 min before fixation. Scale bars, 10 μm (main panels), 1 μm (inset panels). *E* and *F*, HeLa cells expressing LOV2-BAX(ER) and the organelle damage reporters for mitochondria (AIF-mCherry-FRB and FKBP-Halo) (*E*) or for lysosomes (LAMP1-RFP and Halo-Galectin 3) (*F*). Cells were treated with Q-VD-Oph, HaloTag SaraFluor 650T ligand, and the heterodimerizer (only for *E*), stimulated with *blue* light for 60 min, and subsequently incubated for 30 min before fixation. Scale bars, 10 μm (*main panels*), 1 μm (*inset panels*).

Finally, we evaluated the organelle specificity of these tools. To this end, LOV2-BAX constructs were co-expressed with rupture reporters for non-target organelles. LOV2-BAX(mito) did not induce translocation of FKBP-Halo to the ER (Fig. 7*A*) or translocation of Gal3 to lysosomes (Fig. 7*B*), confirming that LOV2-BAX(mito) is specific to mitochondria. Similarly, LOV2-BAX(lyso) did not cause mitochondrial (Fig. 7*C*) and ER rupture (Fig. 7*D*), and LOV2-BAX(ER) did not cause mitochondrial (Fig. 7*E*) and lysosomal rupture (Fig. 7*F*). These results confirm that LOV2-BAX constructs specifically rupture their targeted organelles.

Thus, these data suggest that LOV2-BAX enables blue light-dependent rupture of the specific organelle membranes, including mitochondria, lysosomes, and the ER.

## Discussion

In this study, we developed and validated a set of optogenetic tools to induce membrane rupture of various organelles. Membrane-inserted BAX can be used to induce membrane rupture regardless of the organelle type. In conjunction with photosensitive LOV2, the membrane-rupture activity of BAX is successfully regulated in a blue light-dependent manner. This new technique enables the spatiotemporal induction of membrane ruptures in specific organelles, including mitochondria, lysosomes, and the ER.

Previous studies have reported that tethering of cytoplasmic BAX to mitochondria *via* the optogenetic CRY2 and CIB1 tags can be used to induce the rupture of the OMM (16, 17). Similar results were obtained using the FKBP and FRB tags in the present study (Fig. 2*B*). However, this “tethering strategy” failed to rupture the lysosomal membrane (Fig. 2*C*). This might be because simply tethering the BAX TMD to lysosomes is insufficient for its insertion into the lysosomal membrane, as lysosomes are not the original destination of BAX. Therefore, we modified its TMD to change the localization of “membrane-inserted” BAX, enabling the regulation of pore-forming activity by using LOV2. This strategy has expanded the types of organelles that can be ruptured by BAX. It remains to be determined whether LOV2-BAX can induce membrane rupture in other organelles, such as peroxisomes and the Golgi apparatus.

Although the regulatory mechanism of BAX by LOV2 was not analyzed in this study, LOV2 may inhibit the oligomerization of BAX, which is a key step in forming membrane pores. Oligomerized BAX and BAK accumulate around the edges of pores and are observed as puncta on the mitochondrial membranes (32). Supporting our hypothesis, the localization of EGFP-LOV2-BAX changed from a diffuse pattern to bright spots on organelle membranes upon blue light stimulation (Fig. 3*B*). Further research, including structural analysis, is required to reveal the precise mechanism by which LOV2 regulates BAX.

An inherent limitation of optogenetic tools is that photostimulation can cause photodamage. In this study, cells were exposed to blue light for 60 min. In this condition, we confirmed that BAX-independent membrane rupture was not observed, although cells might have been mildly damaged. To reduce phototoxicity, further optimization, such as shortening the photostimulation duration, is required. The wild-type LOV2 becomes inactivated shortly after photostimulation stops (about 60 s) (33, 34); therefore, continuous stimulation is necessary to sustain BAX activity. LOV2 mutants with prolonged photocycles may be useful in shortening photostimulation duration and reduce unintended phototoxicity.

Unlike chemicals such as LLOMe, optogenetic LOV2-BAX enables spatial regulation of organelle membrane rupture. The induction of membrane rupture at the single organelle level and the analysis of the cellular responses are future challenges. Spatial regulation will also benefit *in vivo* analysis. By regulating both the photostimulated area and the expression pattern of LOV2-BAX, our tools can induce organelle membrane rupture in specific tissues. Investigating *in vivo* responses to organelle membrane rupture will be a critical use of the optogenetic LOV2-BAX system in future studies.

## Experimental procedures

### Antibodies

For immunohistochemistry and immunoblotting, rabbit monoclonal anti-BAX (#14796; Cell Signaling Technology), rabbit monoclonal anti-BAK (ab32371; Abcam), rat monoclonal anti-mCherry (M11217; Thermo Fisher Scientific), mouse monoclonal anti-α-tubulin (T9026; Merck), HRP-conjugated anti-rabbit IgG (111-035-144; Jacson), AlexaFluor 488-conjugated anti-rat IgG (A11006; Thermo Fisher Scientific) antibodies were used.

### Cell lines

HeLa cells were obtained from RIKEN BRC (RCB0007) and cultured in Dulbecco’s modified Eagle’s medium (DMEM) (D6546; Sigma-Aldrich) supplemented with 10% fetal bovine serum (FBS) (S1820-500; Biowest), 50 U/ml penicillin, and 50 μg/ml streptomycin (15070-063; GIBCO), at 37 °C in a 5% CO_2_ incubator.

### Plasmids

Plasmids for transient expression and stable expression in HeLa cells were generated by the Gibson Assembly method as follows. Gene sequences encoding EGFP, LOV2 (35), BAX (17), and the C-terminal regions of rat OMP25, human TMEM106B, and rat Cyb5 were inserted into the retroviral plasmid pMRX-IP (36). Halo, PLAAT3(C113S), Gal3, and FKBP were inserted into the retroviral plasmid pMRX-IB (37). FKBP, FRB, mCherry-BAX(S184E) (17), TOM20, and LAMP1 were inserted into the phCMV plasmid (17). EGFP, BAX, and the C-terminal regions of OMP25, TMEM106B, and Cyb5 were inserted into the lentiviral plasmid pLVX-TetOne-Puro (631849; Clontech) for doxycycline-dependent expression. Mutations in LOV2 or BAX were introduced by site-directed mutagenesis. The amino acid sequences of the constructs used in this study are shown in Table S1.

### Transient expression by lipofection

HeLa cells were transfected with the phCMV plasmid or pMRX-IP plasmid, both of which can be used for transient expression using Lipofectamine 2000 (11668019; Thermo Fisher Scientific).

### siRNA transfection

HeLa cells were transfected with non-target siRNA (final concentration: 7.5 nM in culture medium), BAK siRNA (a mixture of 3 siRNAs, 2.5 nM each), or BAX siRNA (a mixture of 2 siRNAs, 3.75 nM each) using Lipofectamine RNAiMAX (13778075; Thermo Fisher Scientific). Cells were analyzed 48 h after siRNA transfection. The siRNAs listed below, synthesized by NIPPON GENE, were used. BAX siRNAs were designed to target the 3′-untranslated region of endogenous BAX mRNA to avoid inhibiting LOV2-BAX expression.

Non-target: universal negative control siRNA (NIPPON GENE)

siBAX-1_sense: cugccuuggacuguguuuuTT

siBAX-1_antisense: aaaacacaguccaaggcagTT

siBAX-2_sense: gugggcauuuuucuuacuuTT

siBAX-2_antisense: aaguaagaaaaaugcccacTT

siBAK-1_sense: gugguacgaagauucuucaTT

siBAK-1_antisense: ugaagaaucuucguaccacTT

siBAK-2_sense: ggguggcagcccugaacuuTT

siBAK-2_antisense: aaguucagggcugccacccTT

siBAK-3_sense: cccgcuucguggucgacuuTT

siBAK-3_antisense: aagucgaccacgaagcgggTT.

### Stable expression by retrovirus or lentivirus infection

For retrovirus preparation, human embryonic kidney 293T (HEK293T) cells were transiently transfected with a retroviral plasmid, pCG-gag-pol, and pCG-VSV-G, (a gift from Dr T. Yasui, National Institutes of Biomedical Innovation, Health and Nutrition) using Lipofectamine 2000. For lentivirus, HEK293T cells were transiently transfected with a lentiviral plasmid, pCMV-VSV-G (gift from R.A. Weinberg, Whitehead Institute for Biomedical Research, Cambridge, MA), and psPAX2 (gift from D. Trono, Ecole Polytechnique Federale de Lausanne, Lausanne, Switzerland) using Lipofectamine 2000. After cells had been cultured for 3 days, the culture medium containing the virus was collected and passed through a 0.45-μm syringe filter unit. HeLa cells were incubated with the virus for 2 days in DMEM, and stable transformants were selected with 10 μg/ml puromycin (P8833; Sigma-Aldrich), 10 μg/ml blasticidin S (022-18713; Wako Pure Chemical Industries), or 200 μg/ml zeocin (R25001; Thermo Fisher Scientific).

### Doxycycline treatment

To enable doxycycline-dependent BAX mutant expression, HeLa cells were treated with 10 μg/ml doxycycline (D3447; Sigma-Aldrich) with 20 μM quinoline-Val-Asp-difluorophenoxymethylketone (Q-VD-Oph) (S1002; Selleck) and 200 nM Halo SaraFluor 650T Ligand (A308-01; Goryo Chemical) for 24 to 48 h.

### Heterodimerizer treatment

To induce heterodimerization of the FKBP and FRB tags, HeLa cells were treated with 500 nM A/C heterodimerizer (635057; Clontech).

### Blue light stimulation

HeLa cells were cultured in four-compartment glass bottom dishes (627870; Greiner Bio-One). Q-VD-Oph (S1002; Selleck) at 20 μM and Halo SaraFluor 650T Ligand (A308-01; Goryo Chemical) at 200 nM were added to DMEM 10 min before blue light stimulation. Blue light was irradiated from the bottom of the dish for 60 min using an LED plate (LEDA-B LED array and LAD-1 LED array driver; Amuza) at 37 °C, with the following parameters: wavelength of 470 nm, a distance of 5 mm between the LED plate and the dish, a voltage of 13.5 V, an estimated luminosity level of 13.5 mW/cm^2^, in constant mode. Following stimulation, HeLa cells were incubated for 30 min in the dark. To analyze lysosomal pH and calcium leakage from the ER, Lysotracker Red DND-99 (L7528; Thermofisher) at 200 nM and Rhod-4 AM (21,121; AAT Bioquest) 5 μM, respectively, were added to DMEM after the photostimulation. Then, cells were fixed with 4% paraformaldehyde (PFA) in phosphate-buffered saline (PBS) for 30 min. The PFA was removed, and the cells were washed three times with PBS. For observation of Rhod-4, cells were live imaged.

### Immunocytochemistry

Cells were fixed with 4% (w/v) paraformaldehyde for 20 to 30 min, washed with PBS, and permeabilized with PBS containing 50 μg/ml digitonin or 0.1% Triton X-100. Cells were blocked with 3% (w/v) BSA in PBS. Primary antibodies and corresponding secondary antibodies conjugated with Alexa Fluor dyes (Thermo Fisher Scientific) were diluted in the blocking buffer, and cells were incubated with primary antibody solutions for 2 h, then secondary antibody solutions for 1 h at room temperature.

### Immunoblotting

Cells were lysed in lysis buffer [1% Triton X-100, 20 mM Tris-HCl (pH 7.6), 137 mM NaCl, complete protease inhibitor cocktail (Roche: 11873580001)]. The lysates were centrifuged at 20,000*g* for 5 min at 4 °C, and the supernatants were mixed with SDS sample buffer (6×) [46.7 mm Tris–HCl, pH 6.8, 5% glycerol, 1.67% SDS, 1% β-mercaptoethanol and 0.02% bromophenol blue] and used for immunoblotting. Samples were subjected to sodium dodecyl sulfate polyacrylamide gel electrophoresis (SDS-PAGE) and transferred to a polyvinylidene difluoride (PVDF) membrane (IPVH00010, Millipore) using the Trans-Blot Turbo Transfer System (BioRad). The membranes were blocked with blocking buffer [3% bovine serum albumin, 0.1% Tween 20, 20 mM Tris-HCl (pH 7.6), 137 mM NaCl] and incubated with primary antibodies in the blocking buffer, followed by incubation with HRP-conjugated secondary antibodies. Protein bands were detected using a FUSION SOLO S (Vilber).

### Fluorescence microscopy

Fluorescence microscopy images were acquired using a confocal laser microscope (FV3000; Olympus) equipped with a 60× oil-immersion objective lens (NA = 1.4) (PLAPON60XOSC2; Olympus). Images were captured using FluoView (Olympus). The software tool ImageJ was used for the analysis of the captured images.

### Correlative electron and light microscopy

For observation of mitochondrial morphology, HeLa cells expressing EGFP-LOV2-BAX(mito) and Halo-PLAAT3(C113S) were cultured on gridded coverslip-bottom dishes (TCI-3922-035R-1CS, a custom-made product based on 3922–035, with a cover glass attached in the opposite direction; Iwaki) coated with carbon by a vacuum evaporator (IB-29510VET, JEOL). The cells were stimulated by blue light for 60 min and incubated for an additional 30 min, followed by fixation with freshly prepared 2% PFA with 0.5% glutaraldehyde in 0.1 M phosphate buffer (pH 7.4). After washing with 0.1 M phosphate buffer three times, cells were observed with the FV3000 confocal laser scanning microscope system (Olympus), and then postfixed overnight with 2.5% glutaraldehyde in 0.1 M sodium cacodylate buffer at 4 °C. After washing with 0.1 M sodium cacodylate, cells were treated with ferrocyanide-reduced osmium tetroxide (1% (*w*/*v*) OsO_4_, 1.5% (*w*/*v*) K4[Fe(CN)_6_]) in 0.065 M sodium cacodylate buffer for 2 h at 4 °C, and rinsed five times using Milli-Q water. The samples were then stained with 2% uranyl acetate solution for 1 h and rinsed five times using Milli-Q water. The samples were dehydrated with an ethanol gradient series, covered with an EPON812-filled plastic capsule, which was placed inverted over the sample surface, and polymerized at 40 °C for 12 h and then 60 °C for 48 h. After polymerization, cover glasses were removed by soaking in liquid nitrogen, and the sample block was trimmed to about 150 × 150 μm, retaining the same area from which the fluorescence microscopy image was obtained. Then, serial sections (25 nm thick) were cut, collected on a silicon wafer strip, and observed under a scanning electron microscope (SEM; JEM7900F; JEOL).

### Quantification and statistical analysis

To calculate the percentage of mitochondria-ruptured cells and ER-ruptured cells, cells with colocalization of organelle markers and membrane-damage markers were counted manually by a researcher (who was blind to both the constructs and photostimulation of each sample). For quantification of the number of Halo-Gal3 puncta per cell, the brightness of the Halo signal in each cell was adjusted by the ImageJ brightness filter and then binarized with automatic parameters. Puncta (size = 0.05–0.50 μm^2^, circularity = 0.10–1.00) were extracted using the “Analyze Particles” filter in ImageJ. Differences were statistically analyzed by one-way ANOVA. Statistical analysis was performed using Graph-Pad Prism 8 software.

## Data availability

All data supporting the analyses in the manuscript are available from the corresponding author upon reasonable request.

## Supporting information

This article contains supporting information.

## Conflict of interest

The authors declare that they have no conflicts of interest with the contents of this article.
