# Supplementary material for: Optogenetic tools for inducing organelle membrane rupture[image]
Source: J Biol Chem. 2025 Mar 18;301(4):108421. doi: 10.1016/j.jbc.2025.108421 (PMC12017856; doi:10.1016/j.jbc.2025.108421)
Supplement: Supplementary Figures S1–S3 and Table S1 [file mmc1.pdf]

SUPPORTING FIGURES AND TABLES

Optogenetic tools for inducing organelle membrane rupture

Yuto Nagashima[1], Tomoya Eguchi[1*], Ikuko Koyama-Honda[1], Noboru Mizushima[1*]

[1]Department of Biochemistry and Molecular Biology, Graduate School and Faculty of Medicine, The University of Tokyo, Tokyo, Japan

*Correspondence should be addressed to Tomoya Eguchi (e-mail: t-eguchi@m.u-tokyo.ac.jp), Noboru Mizushima (e-mail: nmizu@m.u-tokyo.ac.jp)

List of Supporting Materials

Figure S1. The C-terminus of SEC61B-mCherry-FRB faces the ER lumen.

Figure S2. Endogenous BAX and BAK are not required for LOV2-BAX activity.

Figure S3. Pore-forming activity of LOV2-BAX is required for its organelle-rupture activity.

Table S1. The amino acid sequences of constructs used in this study

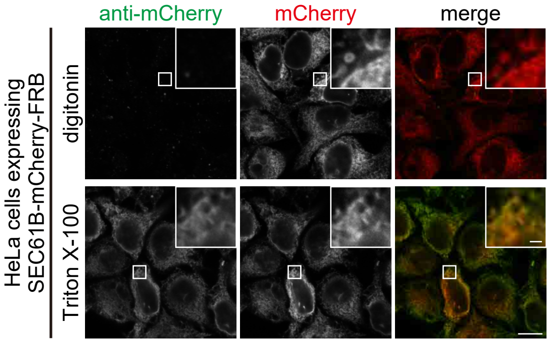

Figure S1. The C-terminus of SEC61B-mCherry-FRB faces the ER lumen.
Hela cells expressing SEC61B-mCherry-FRB were fixed and permeabilized with digitonin (upper panels) or Triton X-100 (lower panels). Cells were stained with anti-mCherry antibody, and the signals of the antibody and mCherry itself were observed. Scale bars, 10 μm (main panels), 1 μm (inset panels).

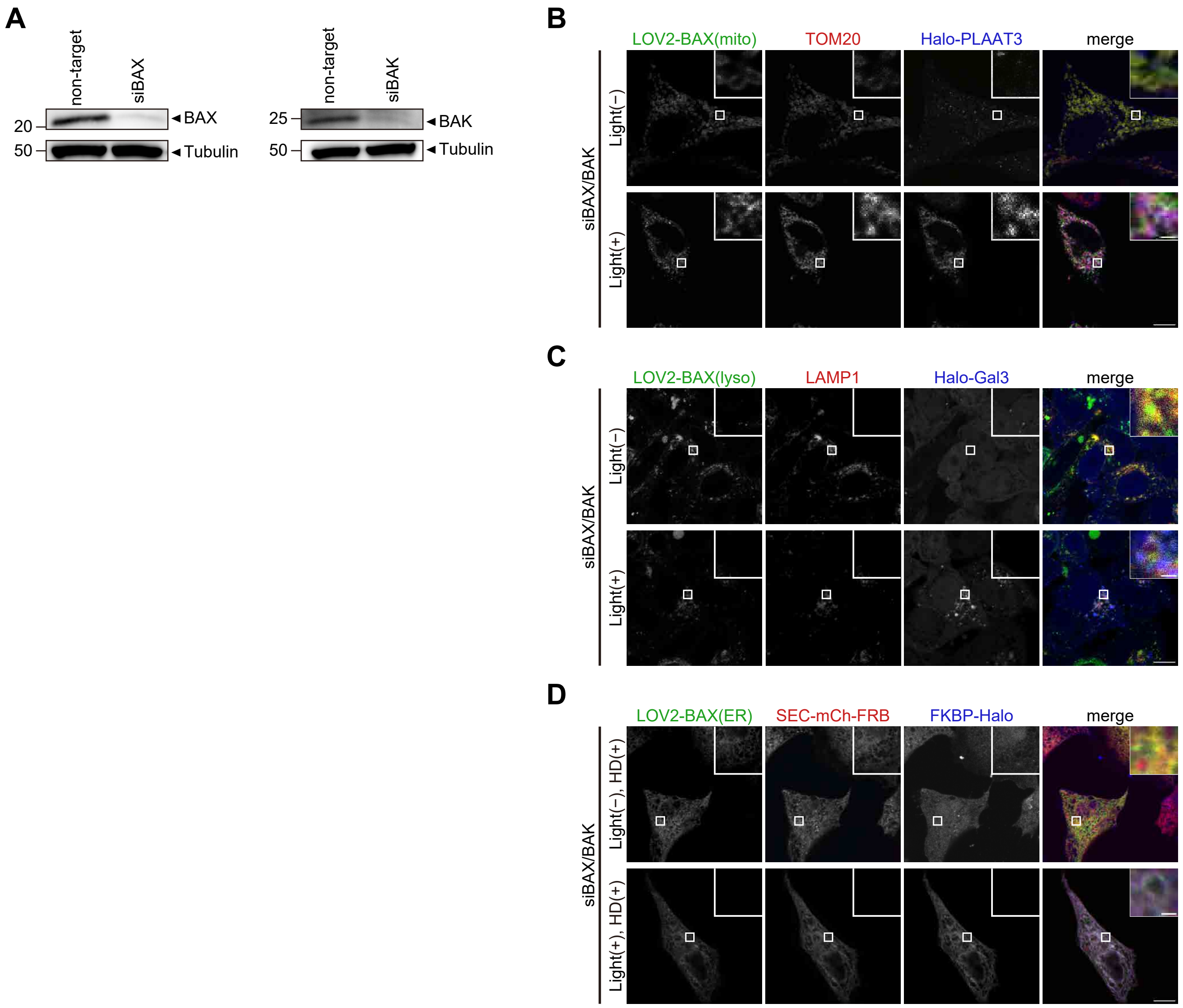

Figure S2. Endogenous BAX and BAK are not required for LOV2-BAX activity.

A, Levels of endogenous BAX and BAK in HeLa cells that were transfected with non-target, BAX or BAK siRNA

B, HeLa cells expressing TOM20-mRFP, Halo-PLAAT3, and GFP-LOV2-BAX(mito) were transfected with BAX and BAK siRNA. Cells were treated with Q-VD-Oph and HaloTag SaraFluor 650T ligand, stimulated with blue light for 60 min, and subsequently incubated for 30 min before fixation. Scale bars, 10 μm (main panels), 1 μm (inset panels).

C, HeLa cells expressing LAMP1-mRFP, Halo-Gal3, and GFP-LOV2-BAX(lyso) were transfected with BAX and BAK siRNA. Cells were treated with Q-VD-Oph and HaloTag SaraFluor 650T ligand, stimulated with blue light for 60 min, and subsequently incubated for 30 min before fixation. Scale bars, 10 μm (main panels), 1 μm (inset panels).

D, HeLa cells expressing SEC61B-mCherry-FRB (SEC-mCh-FRB), FKBP-Halo, and GFP-LOV2-BAX(ER) were transfected with BAX and BAK siRNA. Cells were treated with the heterodimerizer (HD), Q-VD-Oph, and HaloTag SaraFluor 650T ligand, stimulated with blue light for 60 min, and subsequently incubated for 30 min before fixation. Scale bars, 10 μm (main panels), 1 μm (inset panels).

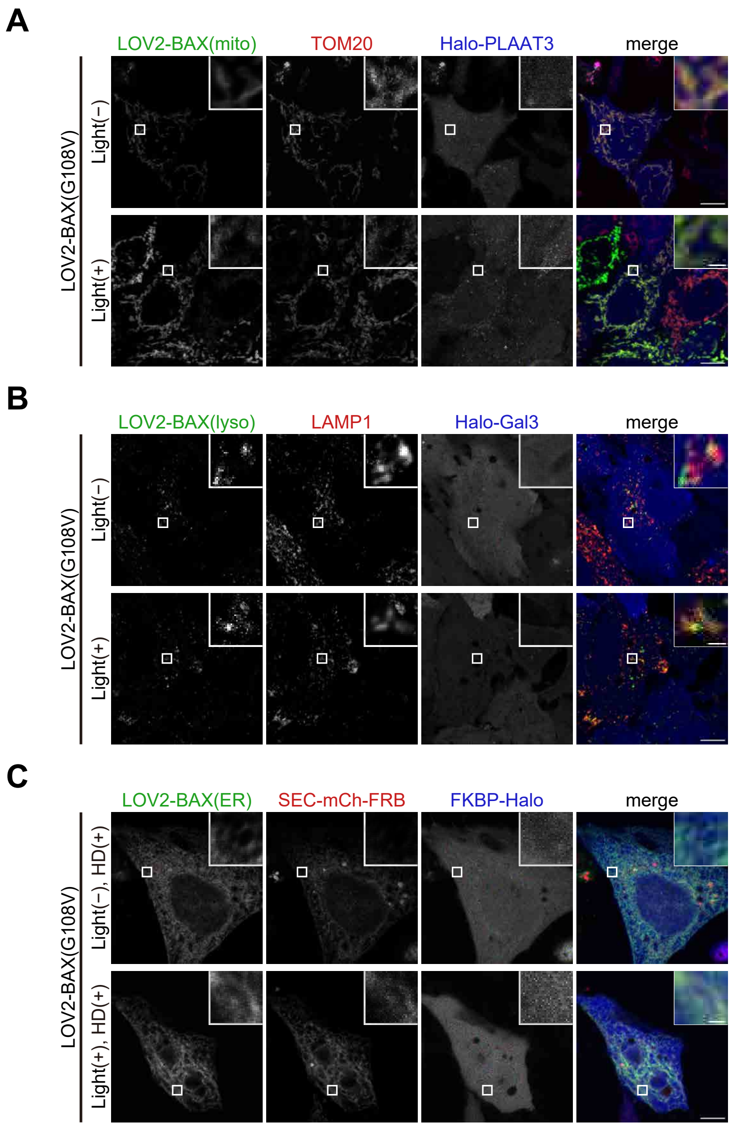

Figure S3. Pore-forming activity of LOV2-BAX is required for its organelle-rupture activity.

A, GFP–LOV2–BAX(mito, G108V), TOM20–mRFP, and Halo–PLAAT3(C113S) were expressed in HeLa cells. Cells were treated with Q-VD-Oph and HaloTag SaraFluor 650T ligand, stimulated with blue light for 60 min, and subsequently incubated for 30 min before fixation. Scale bars, 10 µm (main panels), 1 µm (inset panels).

B, HeLa cells expressing LAMP1-mRFP, Halo-Gal3, and GFP-LOV2-BAX(lyso, G108V) were treated with Q-VD-Oph and HaloTag SaraFluor 650T ligand, stimulated with blue light for 60 min, and subsequently incubated for 30 min before fixation. Scale bars, 10 µm (main panels), 1 µm (inset panels).

C, HeLa cells expressing SEC61B-mCherry-FRB (SEC-mCh-FRB), FKBP-Halo, and GFP-LOV2-BAX(ER, G108V) were treated with the heterodimerizer (HD), Q-VD-Oph, and HaloTag SaraFluor 650T ligand, stimulated with blue light for 60 min, and subsequently incubated for 30 min before fixation. Scale bars, 10 µm (main panels), 1 µm (inset panels).

Name	Details	Used in
EGFP–<br>LOV2–<br>BAX<br>(mito)	MVSKGEELFTGVVPILVELDGDVNGHKFSVSGEGEGDATYGKLTLKFICTTGKLPVPWPTLVTTLTYGVQCFSR<br>YPDHMKQHDFFKSAMPEGYVQERTIFFKDDGNYKTRAEVKFEGDTLVNRIELKGIDFKEDGNILGHKLEYNYN<br>SHNVYIMADKQKNGIKVNFKIRHNIEDGSVQLADHYQQNTPIGDGPVLLPDNHYLSTQSALSKDPNEKRDHMV<br>LLEFVTAAGITLGMDELYKGSGSGGMGTSLATTLERIEKNFVITDPRLPDNPIIFASDSFLQLTEYSREEILGRNCRF<br>LQGPETDRATVRKIRDAIDNQTEVTVQLINYTKSGKKFWNLFHLQPMRDQKGDVQYFIGVQLDGTEHVRDAAE<br>REGVMLIKKTAEEIDEAAKELGSGEQPRGGGPTSSEQIMKTGALLLQGFIQDRAGRMGGEAPELALDPVPQDAS<br>TKKLSECLKRIGDELDSNMELQRMIAAVDTDSPREVFFRVAADMFSDGNFNWGRVVALFYFASKLVLKALCTK<br>VPELIRTIMGWTLDFLRERLLGWIQDQGGWDGLLSYFGTPTWQRHRGDGEPSGVPVAVVLLPVFALTLVAVWAF<br>VRYRKQL*<br><br>EGFP, LOV2, BAX, OMP25, linker	Fig. 3
EGFP–<br>LOV2–<br>BAX<br>(ER)	MVSKGEELFTGVVPILVELDGDVNGHKFSVSGEGEGDATYGKLTLKFICTTGKLPVPWPTLVTTLTYGVQCFSR<br>YPDHMKQHDFFKSAMPEGYVQERTIFFKDDGNYKTRAEVKFEGDTLVNRIELKGIDFKEDGNILGHKLEYNYN<br>SHNVYIMADKQKNGIKVNFKIRHNIEDGSVQLADHYQQNTPIGDGPVLLPDNHYLSTQSALSKDPNEKRDHMV<br>LLEFVTAAGITLGMDELYKGSGSGGMGTSLATTLERIEKNFVITDPRLPDNPIIFASDSFLQLTEYSREEILGRNCRF<br>LQGPETDRATVRKIRDAIDNQTEVTVQLINYTKSGKKFWNLFHLQPMRDQKGDVQYFIGVQLDGTEHVRDAAE<br>REGVMLIKKTAEEIDEAAKELGSGEQPRGGGPTSSEQIMKTGALLLQGFIQDRAGRMGGEAPELALDPVPQDAS<br>TKKLSECLKRIGDELDSNMELQRMIAAVDTDSPREVFFRVAADMFSDGNFNWGRVVALFYFASKLVLKALCTK<br>VPELIRTIMGWTLDFLRERLLGWIQDQGGWDGLLSYFGTPTWQPSETLITTVESNSSWWTNWVIPAISALVVAL<br>MYRLYMAED*<br><br>EGFP, LOV2, BAX, CYB5, linker	Fig. 6
EGFP–<br>LOV2–<br>BAX<br>(lyso)	MVSKGEELFTGVVPILVELDGDVNGHKFSVSGEGEGDATYGKLTLKFICTTGKLPVPWPTLVTTLTYGVQCFSR<br>YPDHMKQHDFFKSAMPEGYVQERTIFFKDDGNYKTRAEVKFEGDTLVNRIELKGIDFKEDGNILGHKLEYNYN<br>SHNVYIMADKQKNGIKVNFKIRHNIEDGSVQLADHYQQNTPIGDGPVLLPDNHYLSTQSALSKDPNEKRDHMV<br>LLEFVTAAGITLGMDELYKGSGSGGMGTSLATTLERIEKNFVITDPRLPDNPIIFASDSFLQLTEYSREEILGRNCRF<br>LQGPETDRATVRKIRDAIDNQTEVTVQLINYTKSGKKFWNLFHLQPMRDQKGDVQYFIGVQLDGTEHVRDAAE<br>REAVMLIKKTAEEIDEAAKELGSGEQPRGGGPTSSEQIMKTGALLLQGFIQDRAGRMGGEAPELALDPVPQDAS<br>TKKLSECLKRIGDELDSNMELQRMIAAVDTDSPREVFFRVAADMFSDGNFNWGRVVALFYFASKLVLKALCTK<br>VPELIRTIMGWTLDFLRERLLGWIQDQGGWDGLLSYFGTPTWQRPRRTKLYVMASVFVCLLLSGLAVFFLFPRSI<br>DVKYIGVKSAYVSYDVQKRTIYLNITNTLNITNNNYYSVEVENITAQVQFSKTVIGKARLNNISIIGPLDMKQIDY<br>TVPTVIAEEMSYMYDFCTLISIKVHNIVLMMQVTVTTTYFGHSEQISQERYQYVDCGRNTTYQLGQSEYLNVLQ<br>PQQ*<br><br>EGFP, LOV2, BAX, TMEM106B, linker	Fig. 5

Table S1. The amino acid sequences of constructs used in this study
